# Supplementary material for: The Construction and Exploration of a Comprehensive MicroRNA Centered Regulatory Network in Foxtail Millet (Setaria italica L.)
Source: Front Plant Sci. 2022 May 6;13:848474. doi: 10.3389/fpls.2022.848474 (PMC9121102; doi:10.3389/fpls.2022.848474)
Supplement: Supplementary file 6 [file Data_Sheet_6.zip › Sit-miRNovel4_Seita.9G371100.1_550_TPlot.pdf]

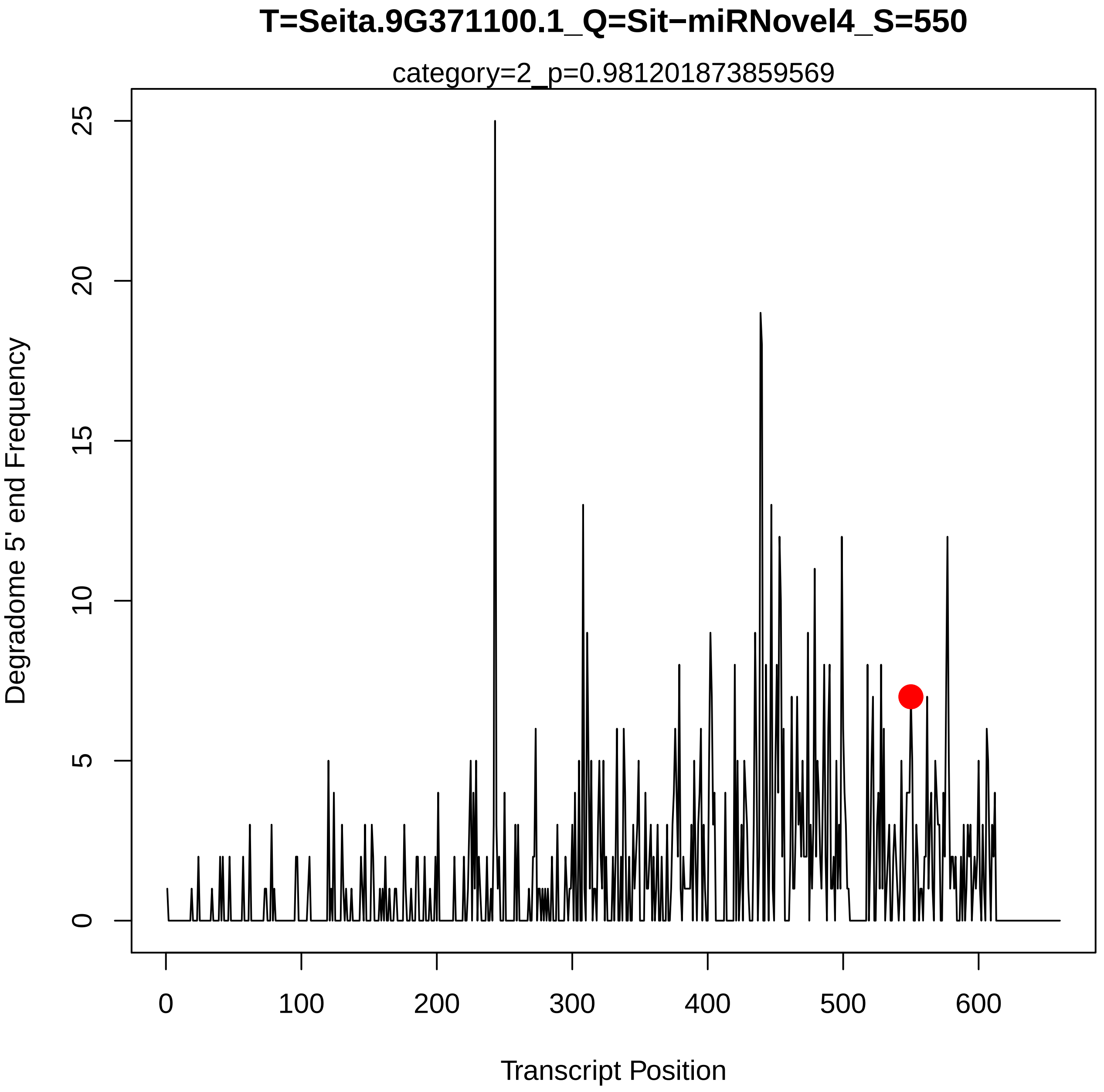
T=Seita.9G371100.1_Q=Sit−miRNovel4_S=550
category=2_p=0.981201873859569
Degradome 5' end Frequency
0
5
10
15
20
25
0
100
200
300
400
500
600
Transcript Position
